# Effect of plasma-derived extracellular vesicles on angiogenesis and the ensuing proliferative diabetic retinopathy through a miR-30b-dependent mechanism

**DOI:** 10.1186/s13098-022-00937-3

**Published:** 2022-12-10

**Authors:** Ping Wang, Chengqian Li, Yujie Deng, Qing Yu, Xuxia Meng, Tao Jiang, Qing Wang, Yudong Fu

**Affiliations:** 1 Department of Endocrinology, The Affiliated Hospital of Qingdao University, Qingdao, 266003 People’s Republic of China; 2 Department of Ophthalmology, The Affiliated Hospital of Qingdao University, No. 16, Jiangsu Road, Qingdao, 266003 Shandong People’s Republic of China

**Keywords:** Proliferative diabetic retinopathy, Plasma, Extracellular vesicles, microRNA-30b, SIRT1, VEGF, Angiogenesis

## Abstract

**Background/purpose:**

Proliferative diabetic retinopathy (PDR) is a major diabetic microvascular complication, characterized by pathological angiogenesis. This study sets out to investigate the potential molecular mechanism in the angiogenesis during PDR.

**Methods:**

The expression of microRNA-30b (miR-30b) was quantified in a streptozotocin (STZ)-induced mouse model of PDR. The binding affinity between SIRT1 and miR-30b was then identified and validated. After transduction with In-miR-30b or combined with sh-SIRT1, high-glucose (HG)-induced retinal microvascular endothelial cells (RMECs) were co-cultured with extracellular vesicles (EVs) derived from the plasma of PDR mice (plasma-EVs). The proliferation and angiogenesis of RMECs were then detected in vitro.

**Results:**

miR-30b expression was upregulated in the retinal tissue of PDR mice. SIRT1 was a target gene of miR-30b and under the negative regulation by miR-30b in RMECs. In contrast, inhibition of miR-30b resulted in elevated SIRT1 expression, thus alleviating the angiogenesis of RMECs. miR-30b was enriched in the plasma-EVs and could be delivered to RMECs, in which miR-30b exerted pro-angiogenic effects. Furthermore, inhibition of miR-30b arrested the progression of PDR in mice by promoting the expression of SIRT1.

**Conclusion:**

Collectively, the present study pinpointed the involvement of miR-30b delivered by plasma-EVs in PDR angiogenesis, thus laying the basis for the development of novel therapeutic targets for the treatment of PDR.

**Supplementary Information:**

The online version contains supplementary material available at 10.1186/s13098-022-00937-3.

## Background

Proliferative diabetic retinopathy (PDR) is more prevalent in individuals with mild diabetic retinopathy (DR) at baseline than those with no DR at baseline [1]. PDR is the more advanced form of DR, characterized by pathological angiogenesis or preretinal/vitreous haemorrhages [2, 3]. Laser photocoagulation, inherently a destructive procedure, is still a mainstay treatment modality for the populations succumbed to PDR [4]. Although significant advancements have been made in the disease etiology and pathogenesis, molecular knowledge on PDR remains incomplete owing to the lack of available ideal clinical samples and experimental research models [5]. Thus, deeper understanding into the processes which drive PDR progression is critical.

Extracellular vesicles (EVs) are membrane vesicles that are released by most cells into the surrounding extracellular environment and can be divided into different subgroups: apoptotic bodies, microvesicles, and exosomes [6]. Plasma-derived EVs (plasma-EVs) have been confirmed to contribute to endothelium damage and the progression of DR [7], which is greatly due to the fact that EVs can transfer bioactive cargoes such as DNA, proteins/peptides, messenger RNAs (mRNAs), microRNAs (miRNAs or miRs), lipids, and organelles to recipient cells [8]. Recently published data have suggested a role for miR-30b-5p as a potential biomarker for the onset of DR [9]. In addition, under high glucose (HG) conditions, miR-30-3p can limit proliferation and migration of human retinal microvascular endothelial cells (RMECs) [10]. Moreover, miR-30 has been largely reported to target Sirtuin 1 (SIRT1) and negatively regulates its expression [11–13]. SIRT1, implicated in the regulation of many cellular functions and transcription of genes, has the potential to maintain retinal vascular and neuronal homeostasis, and aid in the prevention of the development of DR at its early stage [14]. Enhanced expression of SIRT1 has been shown to reduce HG-induced angiogenesis in human retinal endothelial cells in the condition of DR [15]. The aforementioned information allowed us to speculate and then extensively validate the potential role of miR-30b transferred by plasma-EVs in the angiogenesis of retinal microvascular endothelial cells (RMECs) and the resultant PDR progression by targeting SIRT1.

## Materials and methods

### Ethics statement

The current study was ratified by the Animal Ethics Committee of The Affiliated Hospital of Qingdao University and performed based on the Guide for the Care and Use of Laboratory Animals published by the US National Institutes of Health. Due care was taken to reduce the animals’ suffering.

### Establishment of mouse PDR models

Male C57BL/6 mice (6–8 weeks old, weighing 18–22 g, Hunan SJA Laboratory Animal Co., Ltd., Hunan, China) were housed under specific pathogen-free conditions with constant humidity (45–50%) and constant temperature (25–27 °C) for 1 week, with a 12-h light/dark cycle to acclimatize the experimental environment. Mice were fasted for 12 h before administration, and were given ad libitum access to food and water at other times.

Mice were randomized into control group (n = 6) and streptozotocin (STZ) group (n = 24). After fasting for 12 h, the baseline blood glucose levels and body weight of mice were measured. The mice in the STZ group were injected intraperitoneally with STZ (55 mg/kg, S0130, Sigma-Aldrich Chemical Company, St Louis, MO, STZ was dissolved in 0.1 mM sterile sodium citrate buffer before each injection) for 5 consecutive days [14, 16]. The control mice were injected intraperitoneally with the same amount of normal saline. The serum glucose concentration was measured 7 days after the last injection, and the non-fasting blood glucose level was quantified using a commercially available blood glucose meter (HEA-215, Omron, USA). Only the mice with high glucose concentration > 16.7 mmol/L were confirmed as diabetic mice [17]. The body weight and blood glucose of these mice were monitored every 2 weeks during the induction of diabetes. At 4 weeks after STZ injection, 18 diabetic mice were injected with lentivirus carrying inhibitor-negative control (In-NC) + short hairpin RNA (sh)-NC, In-miR-30b + sh-NC and In-miR-30b + sh-SIRT1 via tail vein, twice a week. Eight weeks after STZ injection, all mice were anesthetized by intraperitoneal injection of 1% sodium pentobarbital (30 mg/kg). Abdominal aortic blood samples from control and STZ mice (n = 6) were taken for the extraction of plasma-EVs. Afterwards, the mice were dissected, with the eyes removed, fixed with 4% paraformaldehyde or stored in liquid nitrogen for subsequent detection.

### Optical coherence tomography (OCT)

Eight weeks after STZ injection, OCT was performed on all mice. The mice were weighed and anesthetized and subjected to eye dilation using 0.5% tonyamide solution, followed by operation utilizing a spectral domain-OCT (Spectralis OCT, Heidelberg Engineering, Heidelberg, Germany) (from the posterior pole to the central part of the mouse eye). Finally, the Image-Pro Plus 6.0 analysis software was adopted for evaluation of the retinal thickness.

### Retinal trypsin digestion (RTD) assay

Under aseptic conditions, the retina was incubated with 0.1% Tris buffer (pH 7.8) containing 3% trypsin. The tissue beginning to show signs of disintegration indicated the accomplishment of the trypsin digestion (approximately 1.5 h). The trypsin was carefully removed and the tissues were rinsed with PBS buffer. The internal restriction membrane was peeled off with tweezers, using scissors if necessary, and shaken gently for 5 min. PBS was then removed with a pipette under a microscope to avoid damaging the retina. Repeated water washing (5 min each time) was conducted to separate the vascular network from the adherent retinal tissue. The washing was completed when there was almost no residue in the water. Thereafter, the tissue was stained with periodic acid-Schiff (PAS) and observed under an optical microscope (XP-330, Shanghai BINGYU optical instrument Co., LTD), with 6 random fields of view selected from each retinal tissue to quantify acellular blood vessels and pericytes.

### Hematoxylin–eosin (HE) staining

The eyeball was fixed in 4% paraformaldehyde, paraffin-embedded, cut into 4-µm-thick sections which were subjected to H&E staining (Solarbio, Beijing, China) [18]. All sections were made in the same direction with the cross line always passed through the optic nerve head, and the area was quantified at a certain distance from the optic nerve head. Observation was started under an optical microscope (XP-330, Bingyu). Image-Pro Plus 6.0 software was operated for evaluation of retinal thickness.

### Isolation and culture of mouse RMECs

The retinal tissue was immersed in PBS (15140148, Gibco, Thermo Fisher Scientific Inc., Waltham, MA) containing 5% penicillin-streptomycin for 5 min, and transferred to Dulbecco’s modified Eagle’s medium (DMEM) appended to 10% PBS to remove vitreous. The retinal tissue was cut into small pieces and digested with 1 g/L type II collagenase (1148090, Sigma-Aldrich) at 37 °C for 30 min. Cell suspension was filtered through a 70 μm filter and cultured in endothelial cell medium (ECM; 1001, ScienCell Research Laboratories, San Diego, CA), which was changed every 2–3 days. The cells were treated with HG (30 mM; G8270-100G, Sigma-Aldrich) for 24 h to simulate endothelial cell damage in vitro.

### Lentivirus transduction

RMECs were seeded in a 6-well plate (4 × 10^5^ cells/well) and cultured in an incubator for 24 h. Cells were then transduced with lentivirus (Shanghai GenePharma Co., Ltd., Shanghai, China) carrying NC (Ad-NC), miR-30b (Ad-miR-30b), In-NC, In-miR-30b, In-NC + sh-NC, In-miR-30b + sh-NC and In-miR-30b + sh-SIRT1. The dose of injected lentivirus in mice was 100 µL. The titer of virus was 1 × 10^9^ TU/mL. After 72 h of transduction, the cells were cultured in medium replenishing puromycin (4 µg/mL) for at least 2 weeks. Puromycin-resistant cells were expanded in a medium replenishing 2 µg/mL puromycin for 9 days, and then cultured in a puromycin-free medium to obtain stably expressed cells.

### 5-ethynyl-2′-deoxyuridine (EdU) assay

RMECs were seeded in 24-well plates (1 × 10^5^ cells/mL), fixed with 4% paraformaldehyde for 30 min, and treated with 0.5% Triton X-100 at ambient temperature (15 min). Following reaction with 100 µL of 1×Apollo® reaction mixture (#C10310-1, Guangzhou RiboBio Co., Ltd., Guangzhou, China) for 30 min, the cells were then stained with 5 µg/mL Hoechst 33342 (#62249, Thermo Fisher Scientific) for 10 min, followed by imaging utilizing a fluorescence microscope (DM1000, Leica, Wetzlar, Germany).

### Vessel-like tube formation in vitro

Matrigel (356234; Shanghai Shanlan Biotechnology Co., Ltd., Shanghai, China) was first melted. A pre-chilled micropipette was used to quickly add 70 µL of yellow gel-like liquid to a pre-chilled 96-well plate for maintaining at 37 °C for about 30 min to solidify the matrigel. After transfection, the cells were starved in serum-free medium for 1 h, and suspended in DMEM. The cell suspension containing 1 × 10^5^ cells/mL was seeded into the pre-coated plate, and the cell culture medium of the corresponding group was added to each well, followed by incubation for 18 h and photograph under an inverted microscope (IXplore Standard, Olympus, Japan).

### Dual luciferase reporter assay

With the help of pGL3-basic vector (Promega Corp., Madison, Wisconsin), the firefly luciferase reporter vector pGL3-basic-SIRT1-3′-untranslated region (UTR)-wild type (WT) (SIRT1-WT) and the pGL3-basic-SIRT1-3′-UTR-mutant type (MUT) (SIRT1-MUT) was constructed. HEK-293T cells (American Type Culture Collection, Manassas, VA) and RMECs were seeded in a 24-well plate (1 × 10^4^ cells/well), and incubated for 24 h. Under 50–60% confluence, cells were co-transfected with NC mimic or miR-30b mimic (GenePharma) and SIRT1-WT or SIRT1-MUT using Lipofectamine 2000 reagent. All cells were co-transfected with 10 ng pRL-TK renilla luciferase. After 24 h of transfection, the cell lysate was collected to detect the luciferase activity. By referring to the dual luciferase reporter assay system (E1910, Promega), the relative luciferase activity was measured and normalized to renilla luciferase activity.

### RNA isolation and quantitation

Total RNA was extracted from cells and tissues with TRIzol reagents (Invitrogen Inc., Carlsbad, CA, USA). Following concentration determination, the RNA was reversely transcribed into complementary DNA (cDNA) utilizing PrimeScript™ RT reagent kit with gDNA Eraser kit (RRO37A, TaKaRa, Japan; for mRNA detection) and polyA tailing reverse transcription kit (B532451, Shanghai Sangon biotech company) (for miR-30b detection). RT-qPCR was processed utilizing SYBR Premix Ex TaqTM (Tli RNaseH Plus) kit (RR820A, TaKaRa) on an ABI 7500 instrument (Thermo Fisher Scientific). mRNAs were normalized to GAPDH while miR-30b to U6, respectively. The primer sequences are shown in Additional file 1: Table S1. The fold changes were calculated employing relative quantification (the 2^−ΔΔCt^ method).

### Western blot analysis

Total protein was extracted from tissues or cells with PMSF and protease inhibitor, with the concentration determined by a bicinchoninic acid kit (23227, Thermo Fisher Scientific). The protein was then separated using polyacrylamide gel electrophoresis and transferred onto polyvinylidene fluoride membranes. The membrane was blocked using 5% bovine serum albumin for 1 h and underwent overnight incubation at 4 °C with primary antibodies against mouse anti-SIRT1 (1:1000, #8469, Cell Signaling Technologies, Beverly, MA), mouse anti-VEGF (1:1000, sc-7269, Santa Cruz Biotechnology, Inc., Santa Cruz, CA), and rabbit anti-GAPDH (1:1000, #2118, Cell Signaling Technologies). The next day, the membrane was reacted with HRP-labeled secondary antibody goat anti-mouse (1:10000, BA1050, Boster Biological Technology Co., Ltd., Wuhan, Hubei, China) or goat anti-rabbit IgG (1:10000, BA1054, Boster) for 1 h. The membrane was developed using developer (NCI4106, Pierce, Rockford, IL) and band intensities were quantified employing ImageJ 1.48u software (Bio-Rad, Hercules, CA), with GAPDH serving as a loading control.

### Isolation and identification of EVs

EV Extraction Kit (4484450, Invitrogen Life Technologies, CA) was used to extract plasma-EVs. The plasma samples stored at − 80 °C were taken out, and placed in a 37 °C water bath until completely melted. The samples were put on ice, and centrifuged at 2000×*g* for 20 min to remove cells and debris. The supernatant was harvested and transferred to a new centrifuge tube using a pipette, followed by centrifugation at 10,000×*g* for 20 min at ambient temperature to remove debris. The supernatant was removed into a new centrifuge tube, which was placed on ice for separation. The obtained precipitate was EVs and stored at − 80 °C.

The morphology of EVs was then observed under a transmission electron microscope (TEM; JEM-2000EX; JEOL, Tokyo, Japan). Nanoparticle tracking analyzer (NS300, Malvern Instruments, Malvern, UK) was used to measure the size distribution of EVs. The expression of EV markers (CD63, CD81, Alix and Calnexin) was detected with the help of western blot analysis, with the used antibodies including anti-CD63 (1:1000, 25682-1-AP, Proteintech ProteinTech Group, Chicago, IL), anti-CD81 (1:1000, 66866-1-Ig, Proteintech), Alix (1:1000, 12422-1-AP, Proteintech) and Calnexin (1:1000, ab133615, Abcam).

### Fluorescence microscope for the internalization of EVs by RMECs

The purified EVs were incubated with PKH67 (PKH67GL, Sigma-Aldrich) at ambient temperature for 5 min, washed twice with PBS, and centrifuged at 120,000×*g* for 90 min. The labeled EVs were resuspended in basal medium and reacted with RMECs at 37 °C for 12 h. Following two washes with PBS, the cells were treated with 4′,6-diamidino-2-phenylindole (D9542, Sigma-Aldrich) to stain the nucleus for 10 min, followed by observation under an IX53 fluorescence microscope (Olympus, Tokyo, Japan).

### Microarray-based gene expression profiling

PDR-related miRNA expression dataset GSE140959 was retrieved from the Gene Expression Omnibus database, which contained 10 control samples and 11 PDR samples, with the platform files of GPL16384. Differential analysis was started utilizing R language “limma” package with |logFC| > 2 and adj.p.val < 0.05 set as the threshold. False discovery rate method was used to correct the difference *p* value. A heat map of the differentially expressed miRNAs was plotted employing R “pheatmap” package. The starBase and RNA22 databases were selected for prediction of the downstream miRNAs of MIAT. Downstream target genes of miRNA were predicted using the starBase, miRDB, TargetScan, miRmap and PicTar databases. The intersection results were analyzed with the help of the jvenn tool. STRING tool was applied to analyze the interaction between candidate gene encoding proteins, with the interaction network then obtained and visualized by Cytoscape 3.5.1 software. Based on the nodes and edges of the network, the gene with a larger Degree was considered as the Hub gene and used for further study.

### Statistical analysis

Statistical analysis was started utilizing SPSS 21.0 software (IBM Corp. Armonk, NY). The measurement data were described as mean ± standard deviation. Data between two groups were compared employing unpaired *t*-test. Data among multiple groups were assessed by one-way analysis of variance, followed by Tukey’s post hoc tests with corrections for multiple comparisons. *p* < 0.05 considered statistically significant.

## Results

### High expression of miR-30b in the retinal tissue of PDR mice

**Fig. 1:**
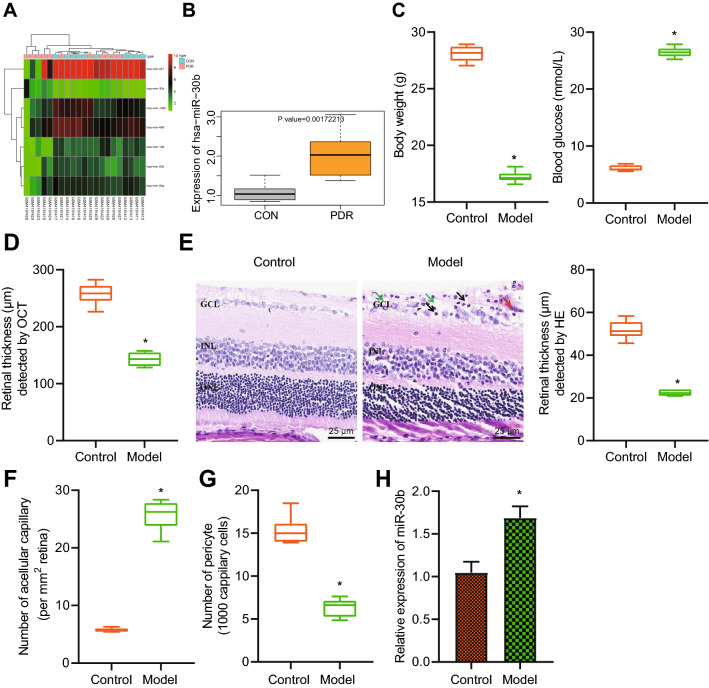
Robustly induced miR-30b in the retinal tissue of PDR mice.** A** A heat map of the expression of significantly differentially expressed miRNAs from PDR-related dataset GSE140959. The color scale from green to red indicates the expression value from small to large. **B** miR-30b expression in the PDR (n = 11) and control samples (n = 10). **C** Body weight and blood glucose level of control and PDR mice at the 8th week. **D**, Total retinal thickness in the circular area of control and PDR mouse optic nerve head determined by OCT. **E** H&E staining analysis of the retinal thickness of control and PDR mice (25 μm). **F** Statistics of the number of acellular capillaries in the retinal tissues of control and PDR mice. **G** Statistics of the number of pericytes in the retinal tissues of control and PDR mice. **H** Expression of miR-30b determined by RT-qPCR in the retinal tissues of control and PDR mice. *p < 0.05, compared with the control mice. n = 6 for control and PDR mice

Differential analysis of the PDR-related miRNA dataset GSE140959 revealed 7 significantly differentially expressed miRNAs (Fig. 1A), of which the differential expression of has-miR-30b was the most significant (log2FC = 1.223220144, *p* = 0.00172213). In addition, miR-30b was found to be significantly highly expressed in PDR samples (Fig. 1B).

To dissect out the mechanism of miR-30b involved in the regulation of PDR, we first established a mouse PDR model by injecting STZ. We identified reduced body weight but increased blood glucose level of the PDR mice (Fig. 1C). The results of OCT and H&E staining showed that the retinal layer of the PDR mice was thinner than that of the control mice (Fig. 1D, E). Moreover, the results of RTD displayed an increase in the acellular capillaries of the PDR mice while pericytes were decreased (Fig. 1F, G). These results indicated that diabetic mice developed retinopathy.

RT-qPCR results suggested that the expression of miR-30b was higher in the retinal tissue of PDR mice than that in the control mice (Fig. 1H). The above results indicated the successful construction of the PDR mouse model and amplified miR-30b expression in the retinal tissue of PDR mice.

### miR-30b targets SIRT1 and inhibits its expression in RMECs

**Fig. 2:**
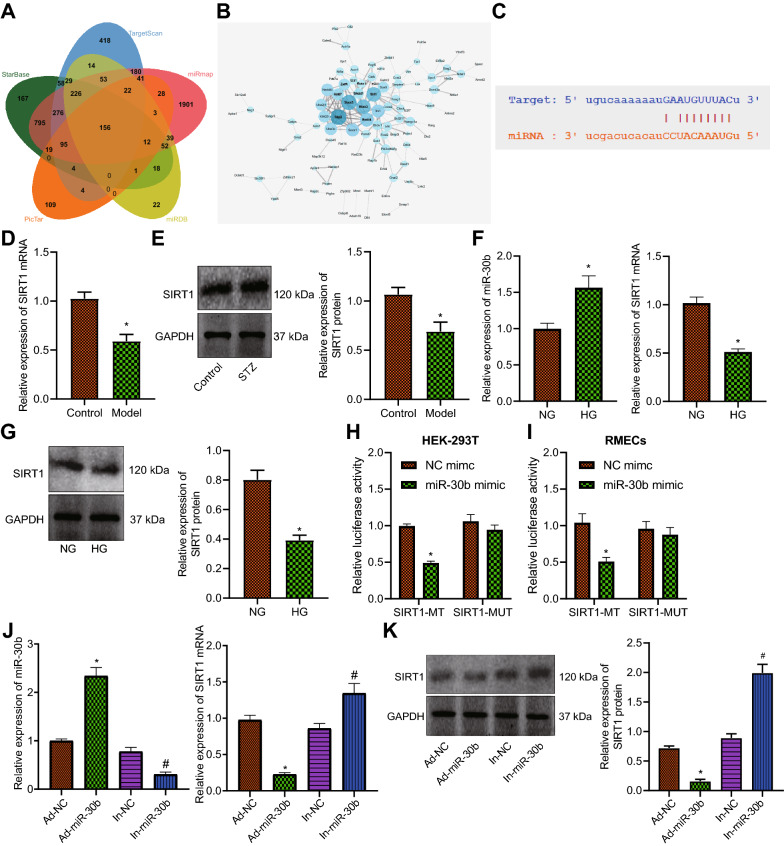
SIRT1 is a target gene of miR-30b. **A** Venn diagram of target mRNAs of miR-30b predicted by the starBase, TargetScan, miRmap, miRDB and PicTar databases. **B** An interaction network of candidate gene encoding proteins. The color of the circle from dark blue to light blue indicates that the Degree value of the gene is from large to small, and the line from dark gray to light gray indicates that the combined score between genes is from large to small. **C** Putative miR-30b binding sites in the 3′UTR of SIRT1 predicted by the starBase database. **D** mRNA expression of SIRT1 determined by RT-qPCR in the retinal tissues of control (n = 6) and PDR mice (n = 6). **E** Western blot analysis of SIRT1 protein in the retinal tissues of control (n = 6) and PDR mice (n = 6). **F** Expression of miR-30b and SIRT1 in NG- and HG-treated RMECs. *NG* normal glucose, *HG* high glucose. **G** Western blot analysis of SIRT1 protein in NG- and HG-treated RMECs. **H** Binding of miR-30b to SIRT1 confirmed by dual luciferase reporter assay in HEK293T cells. **I** Binding of miR-30b to SIRT1 confirmed by dual luciferase reporter assay in RMECs. **J** Expression of miR-30b and SIRT1 determined by RT-qPCR in RMECs transduced with Ad-miR-30b or In-miR-30b. **K** Western blot analysis of SIRT1 protein in RMECs transduced with Ad-miR-30b or In-miR-30b. *p < 0.05, compared with the control mice or NG-treated cells, HEK293T cells and RMECs transfected with NC mimic, or RMECs transduced with Ad-NC. ^#^*p* < 0.05, compared with RMECs transduced with In-NC. Cell experiments were conducted 3 times independently

We then sought to analyze the downstream target of miR-30b in PDR. Venn diagram analysis of the predicted mRNAs of miR-30b by the starBase, TargetScan, miRmap, miRDB and PicTar databases, 156 candidate genes were obtained (Fig. 2A). An interaction network of candidate gene encoding proteins showed that SKP2, SOCS3, RUNX2, SIRT1 and NEDD4 had greater Degree (Fig. 2B). The starBase database predicted the binding sites of miR-30b in the 3’UTR of SIRT1 (Fig. 2C). Additionally, a decline in the SIRT1 expression was found in the retinal tissue of PDR mice (Fig. 2D, E). Meanwhile, SIRT1 expression was reduced while miR-30b expression was increased in HG-induced RMECs (Fig. 2 F, G).

Furthermore, the results of dual luciferase reporter assay indicated that the luciferase activity of SIRT1-WT was reduced following transection with miR-30b mimic while that of SIRT1-MUT remained unchanged (Fig. 2H, I), suggesting that miR-30b can target SIRT1. As depicted in Fig. 2J, K, overexpression of miR-30b in RMECs augmented miR-30b expression but decreased SIRT1 expression; however, opposing trends were detected upon loss-of-function of miR-30b. Therefore, miR-30b could target SIRT1 and consequently repress its expression in RMECs.

### Knockdown of miR-30b inhibits VEGF expression and HG-induced angiogenesis through upregulation of SIRT1

**Fig. 3:**
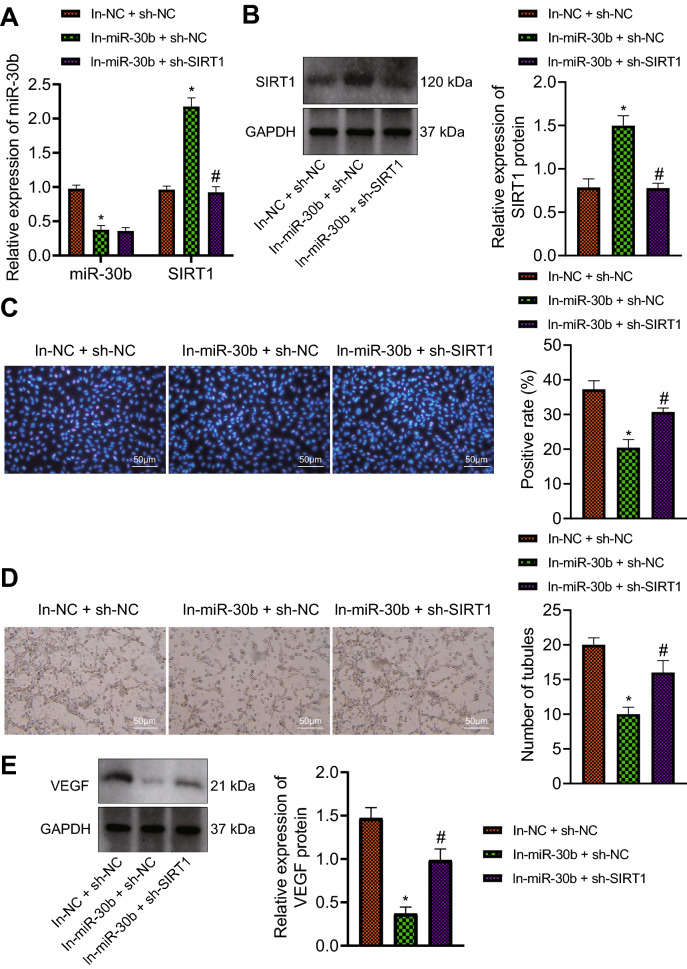
Inhibition of miR-30b alleviates HG-induced angiogenesis through upregulation of SIRT1. RMECs were transduced with In-miR-30b + sh-NC or In-miR-30b + sh-SIRT1.** A** Expression of miR-30b and SIRT1 determined by RT-qPCR in RMECs. **B** Western blot analysis of SIRT1 protein in RMECs. **C** EdU-positive RMECs (50 μm). **D** Angiogenesis of RMECs determined by vessel-like tube formation assay (50 μm). **E** Western blot analysis of VEGF protein in RMECs. *p < 0.05, compared with the RMECs transfected with In-NC + sh-NC. ^#^*p* < 0.05, compared with RMECs transduced with In-miR-30b + sh-NC. Cell experiments were conducted 3 times independently

We further explored whether miR-30b affects the angiogenesis of RMECs by regulating SIRT1. RT-qPCR and Western blot analysis data showed a reduction in the expression of miR-30b and an enhancement in the expression of SIRT1 in RMECs transduced with In-miR-30b + sh-NC while further silencing of SIRT1 failed to alter the expression of miR-30b but downregulated SIRT1 expression (Fig. 3A, B).

In addition, EdU data illustrated that the number of EdU-positive cells was reduced in the absence of miR-30b but it was increased following concomitant silencing of miR-30b and SIRT1 (Fig. 3C). Figure 3D presents inhibition of vessel-like tube formation in RMECs transduced with In-miR-30b + sh-NC, while an opposite result was noted in the presence of In-miR-30b + sh-SIRT1. Meanwhile, miR-30b inhibition in RMECs led to a decline in the protein expression of VEGF, which was negated by additional silencing of SIRT1 (Fig. 3E). The above data indicated that suppression of miR-30b could reduce VEGF expression and HG-induced angiogenesis through upregulation of SIRT1.

### Plasma-EVs deliver miR-30b to promote angiogenesis

**Fig. 4:**
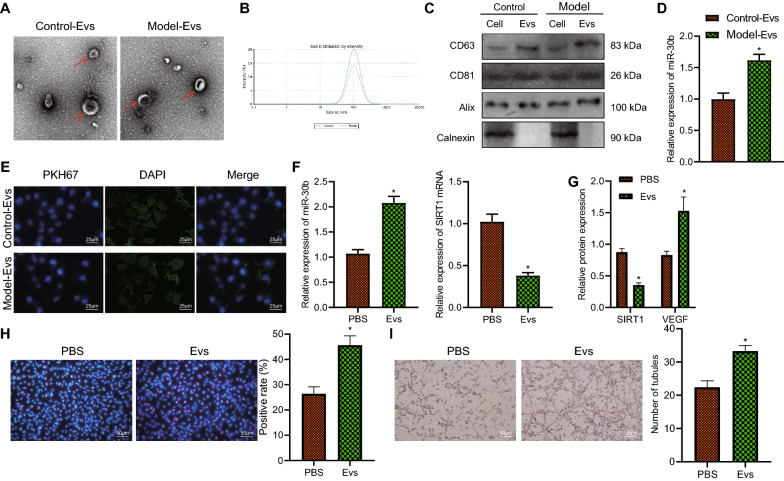
Plasma-EVs transfer miR-30b to RMECs and thus induce angiogenesis.** A** Morphology of the isolated plasma-EVs from control and PDR mice observed under a TEM (100 μm). **B** The particle size of the isolated plasma-EVs from control and PDR mice measured by nanoparticle tracking analyzer. **C** Western blot analysis of EV marker proteins CD63, CD81, Alix, and Calnexin in the isolated plasma-EVs from control and PDR mice. **D** Expression of miR-30b determined by RT-qPCR in the plasma-EVs isolated from control and PDR mice. **E** Internalization of PKH67-labeled EVs by RMECs (25 μm). **F** Expression of miR-30b and SIRT1 determined by RT-qPCR in the RMECs co-cultured with plasma-EVs. **G** Western blot analysis of SIRT1 and VEGF proteins in the RMECs co-cultured with plasma-EVs. **H** EdU-positive RMECs co-cultured with plasma-EVs (50 μm). **I** Angiogenesis of RMECs co-cultured with plasma-EVs determined by vessel-like tube formation assay (50 μm). *p < 0.05, compared with the RMECs treated with PBS. **p < 0.01, compared with the RMECs co-cultured with plasma-EVs from control mice. Cell experiments were conducted 3 times independently

We first isolated plasma-EVs from control and PDR mice. Under a TEM, the isolated plasma-EVs were cup-shaped or oval, with typical EV shape (Fig. 4A), and nanoparticle tracking analyzer revealed that the particle size ranged 30–150 nm (Fig. 4B). The results of western blot analysis exhibited that EV markers CD63, CD81 and Alix were all highly expressed in the isolated EVs, while Calnexin was almost not expressed (Fig. 4C). The expression of miR-30b was detected to be prominently expressed in the plasma-EVs isolated from PDR mice (Fig. 4D).

Co-culture data showed that PKH67-labeled EVs could be internalized by RMECs (Fig. 4E). RT-qPCR results presented higher expression of miR-30b and lower expression of SIRT1 in RMECs co-cultured with EVs than that in the PBS-treated RMECs (Fig. 4F). Western blot analysis results showed reduced protein expression of SIRT1 and increased protein expression of VEGF in RMECs co-cultured with EVs (Fig. 4G). Additionally, the number of EdU-positive cells and vessel-like tube formation of RMECs co-cultured with EVs were increased (Fig. 4H, I). Overall, our findings supported that plasma-EVs could deliver miR-30b to RMECs where it promoted angiogenesis.

### Knockdown of miR-30b arrests the occurrence of PDR in mice by promoting SIRT1 expression

**Fig. 5:**
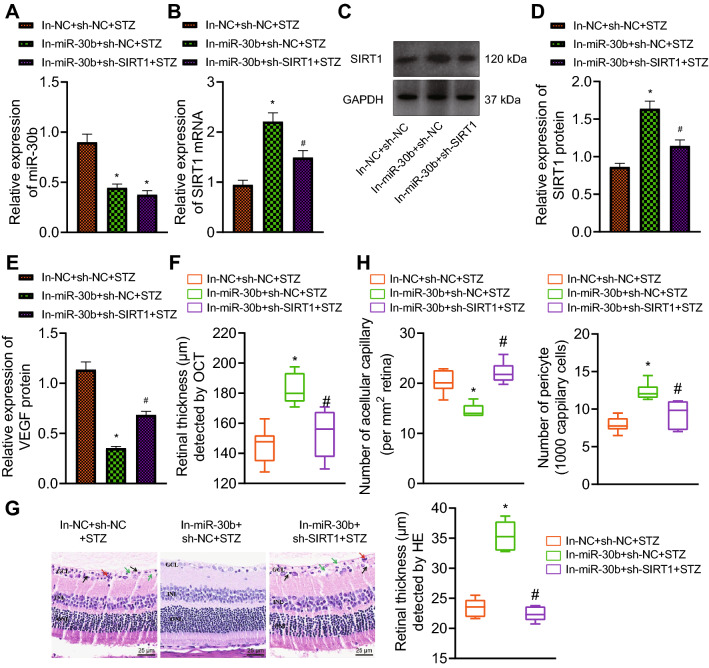
Inhibition of miR-30b prevents PDR in mice by upregulating SIRT1. STZ-induced mice were treated with In-miR-30b + sh-NC or In-miR-30b + sh-SIRT1.** A** Expression of miR-30b determined by RT-qPCR in the retinal tissues of mice. **B** Expression of SIRT1 determined by RT-qPCR in the retinal tissues of mice. **C** Western blot analysis of SIRT1 and VEGF proteins in the retinal tissues of mice. **D** Quantitative analysis of SIRT1 protein expression. **E** Quantitative analysis of VEGF protein expression. **F** Total retinal thickness in the circular area of mouse optic nerve head. **G** H&E staining analysis of the mouse retinal thickness (25 μm). **H** Statistics of the number of acellular capillaries in the mouse retinal tissues and the number of pericytes in the mouse retinal tissues. n = 6 for mice upon each treatment. *p < 0.05, compared with mice treated with In-NC + sh-NC + STZ. ^#^*p* < 0.05, compared with mice treated with In-miR-30b + sh-NC + STZ

Finally, we aimed to determine whether knockdown of miR-30b can inhibit the occurrence of PDR in mice by promoting the expression of SIRT1. As shown in Fig. 5A–E, the expression of miR-30b and VEGF was reduced but that of SIRT1 was elevated in the retinal tissue of PDR mice treated with In-miR-30b + sh-NC, the effect of which was negated by treatment with In-miR-30b + sh-SIRT1.

Additionally, the results of OCT and H&E staining showed that the retinal layer was thickened in the absence of miR-30b while further silencing of SIRT1 reduced the thickness of retinal layer (Fig. 5F, G). The results of RTD assay showed that inhibition of miR-30b led to a decline in the acellular capillaries in the retinal tissue of mice yet an enhancement in the pericytes. In contrast, simultaneous silencing of miR-30b and SIRT1 caused opposite results (Fig. 5H). Cumulatively, knockdown of miR-30b could retard PDR in mice by promoting the expression of SIRT1.

## Discussion

Pathological angiogenesis of the retina in the condition of PDR is a leading irreversible cause of blindness and targeting angiogenesis has been therefore considered a promising therapy to treat PDR [19]. The findings collected from this study supported the promoting effect of miR-30b shuttled by plasma-EVs on the angiogenesis of RMECs and the ensuing progression of PDR by targeting SIRT1, which might shed new light on a novel potentially marker and molecular therapeutic target for PDR.

Our initial results provided evidence suggesting that miR-30b was elevated in the retinal tissue of PDR mice and the HG-induced RMECs. Partially in line with this, miR-30 expression has been confirmed to be upregulated in the diabetic nephropathy group compared with the control group [20]. In addition, miR-30a expression is increased in the vitreous of patients suffering from PDR while its inhibition decreases pathological neovascularization [21]. The current study unveiled that knockdown of miR-30b could diminish VEGF expression and consequently suppressed HG-induced angiogenesis in RMECs. VEGF can regulate vascular and lymphatic growth and its increase is connected to many pathological angiogenesis [22]. Meanwhile, it is associated with many pathologic disorders, including retinopathy, and its inhibition is thus a potential promising strategy to treat these diseases [23]. Previous study demonstrated that elevated miR-30 in endothelial cells could induce angiogenic sprouting in vitro [24]. Thus, it is reasonable to suggest based on the aforementioned findings that miR-30b inhibition has a promising future in pathological angiogenesis during PDR by suppressing VEGF expression.

The subsequent finding in the current study demonstrated that the inhibiting effect of miR-30b knockdown on the angiogenesis of RMECs was related to the upregulation of SIRT1. SIRT1 has been documented to be a target of miR-30c and is under the negative regulation of miR-30c in PC12 cells [25], which is in agreement with our results that miR-30b could target SIRT1 and consequently repress its expression in RMECs. SIRT1 activation is capable of improving DR due to its suppressing effect on the oxidative stress-induced apoptosis and inflammation [26]. Moreover, decreased SIRT1 is detected in the retinal tissues of mice with DR and HG-treated RMECs, while its overexpression greatly represses RMEC proliferation and angiogenesis, thus inhibiting the development of DR [27]. Collectively, these findings illuminate that disruption of miR-30b-mediated inhibition of SIRT1 may be used as a potential novel target for the prevention strategy for HG-induced angiogenesis.

Further analysis exhibited that plasma-EVs transferred miR-30b to RMECs where miR-30b exerted pro-angiogenesis effects. Plasma-EVs isolated from the subjects with DR contribute to pericyte detachment and pericyte/endothelial cell migration, as well as facilitating the formation of vessel-like structure [9]. A recent study has identified that the increased miR-30b-5p in the EVs isolated from diabetic patients with DR can be served as a prognostic biomarker for DR as evidenced by enhanced vessel destabilization and angiogenesis in human microvascular endothelial cells [28]. Meanwhile, inhibition of miR-30 aids in the protection of podocytes from HG-induced cell injury and prevention of diabetic nephropathy progression [29]. Consistently, the present study indicated that knockdown of miR-30b arrested the occurrence of PDR in mice by promoting SIRT1 expression. Therefore, plasma-EVs could package miR-30b and transferred it to RMECs where miR-30b acted as a potential driver to stimulate cell angiogenesis and the consequent PDR progression by downregulating SIRT1.

Although this study further reveals the molecular mechanism of miR-30b promoting PDR, and reveals the biological significance of high expression of miR-30b in PDR patients from the molecular level, there are still many unsolved problems, such as whether SIRT1 is the only target of miR-30b in the process of regulating PDR, and whether Skp2, SOCS3 and Runx2 found by bioinformatic analysis are also regulated by miR-30b, and this is what we are interested in the follow-up. Moreover, whether the detection of miR-30b in plasma EVs can indeed predict the occurrence of PDR is also worth us to continue to explore its potential clinical significance.

**Fig. 6:**
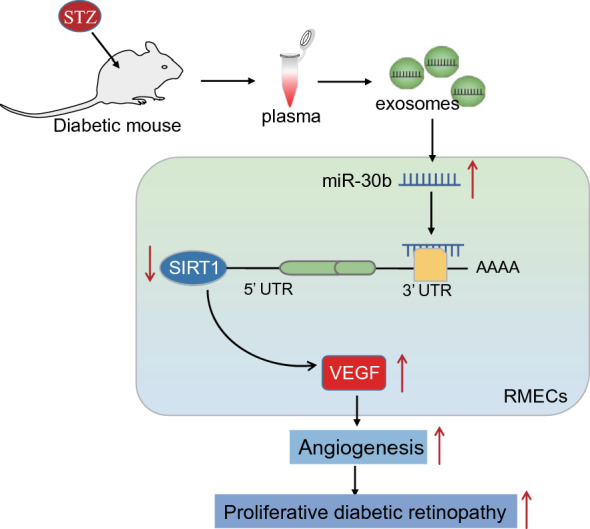
Schematic diagram of the mechanism by which miR-30b shuttled by plasma-EVs affects angiogenesis and PDR progression. Plasma-EVs deliver miR-30b to RMECs where miR-30b targets SIRT1 and inhibits its expression, upregulating VEGF expression and inducing angiogenesis, thereby promoting the occurrence of PDR

In conclusion, our findings reveal that plasma-EVs can potentially deliver miR-30b to RMECs under HG conditions. miR-30b targets SIRT1 and inhibits its expression, resulting in enhanced VEGF expression and angiogenesis, eventually promoting the occurrence of PDR (Fig. 6). Our findings provide mechanistic insight of unrecognized roles of plasma-EV miR-30b targeting SIRT1 in the progression of PDR. The level of miR-30b in plasma-EVs could be used as a potential indicator for molecular diagnosis of PDR. In addition, our study provides a variety of possibilities for the clinical treatment of PDR, including direct targeted inhibition of miR-30b expression, targeted promotion of SIRT1 activity, or specific targeted inhibition of miR-30b in plasma exosomes, or direct inhibition of secretion and internalization of plasma exosomes. However, the clinical value of our study needs the support of more clinical basic research in the future.

## Supplementary Information

**Additional file 1: Table S1. **Primer sequences for reverse transcription quantitative polymerase chainreaction.

## Data Availability

Not applicable.

## Abbreviations

PDR: Proliferative diabetic retinopathy
miR-30b: microRNA-30b
STZ: Streptozotocin
HG: High-glucose
RMECs: Retinal microvascular endothelial cells
DR: Diabetic retinopathy
EVs: Extracellular vesicles
plasma-EVs: Plasma-derived EVs
mRNAs: Messenger RNAs
miRNAs or miRs: microRNAs
HG: High glucose
SIRT1: Sirtuin 1
SPF: Specific pathogen-free
STZ: Streptozotocin
In-NC: Inhibitor-negative control
sh: Short hairpin RNA
OCT: Optical coherence tomography
SD: Spectral domain
RTD: Retinal trypsin digestion
PAS: Periodic acid-Schiff
HE: Hematoxylin-eosin
DMEM: Dulbecco’s modified Eagle’s medium
EdU: 5-Ethynyl-2′-deoxyuridine
UTR: Untranslated region
WT: Wild type
MUT: Mutant type
cDNA: Complementary DNA
TEM: Transmission electron microscope
GEO: Gene Expression Omnibus
FDR: False discovery rate
