# Supplementary material for: Effect of plasma-derived extracellular vesicles on angiogenesis and the ensuing proliferative diabetic retinopathy through a miR-30b-dependent mechanism
Source: Diabetol Metab Syndr. 2022 Dec 10;14:188. doi: 10.1186/s13098-022-00937-3 (PMC9738026; doi:10.1186/s13098-022-00937-3)
Supplement: Supplementary file 1 — Additional file 1: Table S1. Primer sequences for reverse transcription quantitative polymerase chainreaction. [file 13098_2022_937_MOESM1_ESM.docx]

**Table S1.** Primer sequences for reverse transcription quantitative polymerase chain reaction

|  | Sequences |
| --- | --- |
| miR-30b | F:5´-TGTAAACATCCTACACTCAGCT-3´ |
|  | R: Universal reverse primer |
| U6 | F:5´-GCTTCGGCAGCACATATACT-3´ |
|  | R: Universal reverse primer |
| SIRT1 | F:5´-ATGCCAGAGTCCAAGTTTAGAAGAACC-3′ |
|  | R:5´-AAATCCAGATCCTCCAGCACATTCG-3′ |
| GAPDH | F:5´-GCATGGCCTTCCGTGTTC-3′ |
|  | R:5´-GATGTCATCATACTTGGCAGGTTT-3′ |
